# Supplementary material for: Hot spot prediction in protein-protein interactions by an ensemble system
Source: BMC Syst Biol. 2018 Dec 31;12(Suppl 9):132. doi: 10.1186/s12918-018-0665-8 (PMC6311905; doi:10.1186/s12918-018-0665-8)
Supplement: Supplementary file 2 — All relevant MATLAB code. A simple Matlab implement of our predictor. (ZIP 19,742 kb) [file 12918_2018_665_MOESM2_ESM.zip › Additional file 2/Read ASA/ASA website/dbMPIKT/4O27A.html]

 NetSurfP - Protein Surface Accessibility and Secondary Structure Predictions

  

|  |  |  |
| --- | --- | --- |
|  |  | NetSurfP - Protein Surface Accessibility and Secondary Structure Predictions  Technical University of Denmark |

  


---

```
# For publication of results, please cite:
# A generic method for assignment of reliability scores applied to solvent accessibility predictions.
# Bent Petersen, Thomas Nordahl Petersen, Pernille Andersen, Morten Nielsen and Claus Lundegaard
# BMC Structural Biology 2009, 9:51 doi:10.1186/1472-6807-9-51
#
# Column 1: Class assignment - B for buried or E for Exposed - Threshold: 25% exposure, but not based on RSA
# Column 2: Amino acid
# Column 3: Sequence name
# Column 4: Amino acid number
# Column 5: Relative Surface Accessibility - RSA
# Column 6: Absolute Surface Accessibility
# Column 7: Z-fit score for RSA prediction
# Column 8: Probability for Alpha-Helix
# Column 9: Probability for Beta-strand
# Column 10: Probability for Coil
E M  4O27A                  1    0.871 174.187  -1.046   0.000   0.001   0.999
E A  4O27A                  2    0.416  45.832  -1.356   0.003   0.003   0.994
E S  4O27A                  3    0.473  55.471   0.380   0.016   0.005   0.979
B P  4O27A                  4    0.143  20.249  -1.199   0.858   0.002   0.139
E A  4O27A                  5    0.330  36.311  -0.074   0.923   0.002   0.076
E D  4O27A                  6    0.522  75.235   0.595   0.923   0.002   0.076
B I  4O27A                  7    0.073  13.486   0.886   0.970   0.001   0.030
B V  4O27A                  8    0.032   4.934   0.511   0.970   0.001   0.030
E K  4O27A                  9    0.433  89.006   1.720   0.970   0.001   0.030
B N  4O27A                 10    0.260  38.020   1.408   0.970   0.001   0.030
B L  4O27A                 11    0.031   5.731   0.520   0.970   0.001   0.030
B K  4O27A                 12    0.251  51.569   0.816   0.970   0.001   0.030
E E  4O27A                 13    0.504  88.101   0.803   0.970   0.001   0.030
B S  4O27A                 14    0.114  13.396  -0.143   0.970   0.001   0.030
B M  4O27A                 15    0.065  13.047   0.482   0.970   0.001   0.030
E A  4O27A                 16    0.382  42.151   0.634   0.923   0.002   0.076
E V  4O27A                 17    0.336  51.612   0.749   0.858   0.002   0.139
B L  4O27A                 18    0.079  14.428  -0.008   0.782   0.003   0.216
E E  4O27A                 19    0.363  63.451  -0.068   0.622   0.015   0.363
E K  4O27A                 20    0.644 132.450   0.256   0.321   0.003   0.675
E Q  4O27A                 21    0.453  80.959  -0.773   0.109   0.005   0.886
E D  4O27A                 22    0.647  93.262  -0.111   0.053   0.005   0.942
E I  4O27A                 23    0.386  71.392  -1.082   0.053   0.005   0.942
E S  4O27A                 24    0.555  65.069  -1.069   0.109   0.005   0.886
E D  4O27A                 25    0.605  87.224  -1.186   0.321   0.003   0.675
E K  4O27A                 26    0.668 137.305   1.048   0.782   0.003   0.216
E K  4O27A                 27    0.386  79.297   0.544   0.858   0.002   0.139
B A  4O27A                 28    0.222  24.464   0.397   0.923   0.002   0.076
E E  4O27A                 29    0.481  83.996   0.759   0.923   0.002   0.076
E K  4O27A                 30    0.452  92.915   1.524   0.970   0.001   0.030
B A  4O27A                 31    0.081   8.948   0.488   0.970   0.001   0.030
B T  4O27A                 32    0.124  17.185   0.698   0.970   0.001   0.030
E E  4O27A                 33    0.450  78.545   0.768   0.988   0.000   0.012
E E  4O27A                 34    0.329  57.424   0.722   0.988   0.000   0.012
B V  4O27A                 35    0.029   4.380   0.809   0.988   0.000   0.012
B S  4O27A                 36    0.126  14.814   0.287   0.970   0.001   0.030
E K  4O27A                 37    0.508 104.454   1.225   0.970   0.001   0.030
B N  4O27A                 38    0.146  21.433   0.335   0.970   0.001   0.030
B L  4O27A                 39    0.042   7.690   0.492   0.970   0.001   0.030
E V  4O27A                 40    0.376  57.776   1.057   0.970   0.001   0.030
E A  4O27A                 41    0.372  41.016   0.860   0.938   0.007   0.055
B M  4O27A                 42    0.033   6.603   0.463   0.938   0.007   0.055
B K  4O27A                 43    0.216  44.328   1.077   0.938   0.007   0.055
E E  4O27A                 44    0.441  77.043   0.479   0.911   0.033   0.057
B I  4O27A                 45    0.120  22.163   1.090   0.831   0.044   0.125
B L  4O27A                 46    0.043   7.837   0.070   0.701   0.107   0.192
E Y  4O27A                 47    0.289  61.802   0.305   0.307   0.165   0.527
E G  4O27A                 48    0.420  33.062  -0.360   0.053   0.043   0.903
E T  4O27A                 49    0.527  73.026  -0.428   0.018   0.019   0.964
E N  4O27A                 50    0.672  98.337  -0.823   0.016   0.005   0.979
E E  4O27A                 51    0.563  98.409  -1.106   0.053   0.005   0.942
E K  4O27A                 52    0.644 132.471   0.120   0.058   0.017   0.925
E E  4O27A                 53    0.451  78.720   0.009   0.058   0.017   0.925
E P  4O27A                 54    0.471  66.792  -0.077   0.058   0.017   0.925
E Q  4O27A                 55    0.348  62.224   0.666   0.176   0.004   0.820
E T  4O27A                 56    0.490  67.977   0.245   0.923   0.002   0.076
E E  4O27A                 57    0.658 115.005   0.430   0.970   0.001   0.030
B A  4O27A                 58    0.218  24.024   1.123   0.970   0.001   0.030
B V  4O27A                 59    0.050   7.731   0.933   0.988   0.000   0.012
E A  4O27A                 60    0.334  36.774   1.062   0.970   0.001   0.030
E Q  4O27A                 61    0.399  71.351   1.394   0.988   0.000   0.012
B L  4O27A                 62    0.049   8.917   1.025   0.970   0.001   0.030
B A  4O27A                 63    0.057   6.292   0.617   0.988   0.000   0.012
E Q  4O27A                 64    0.351  62.689   1.177   0.970   0.001   0.030
E E  4O27A                 65    0.290  50.698   1.146   0.970   0.001   0.030
B L  4O27A                 66    0.059  10.711   0.717   0.970   0.001   0.030
B Y  4O27A                 67    0.116  24.853   1.058   0.858   0.002   0.139
E N  4O27A                 68    0.459  67.154   0.463   0.782   0.003   0.216
E S  4O27A                 69    0.307  35.992   0.239   0.600   0.003   0.397
E G  4O27A                 70    0.354  27.860  -0.637   0.321   0.003   0.675
B L  4O27A                 71    0.050   9.155   1.214   0.858   0.002   0.139
B L  4O27A                 72    0.041   7.525   0.584   0.975   0.003   0.022
B S  4O27A                 73    0.214  25.034   0.967   0.975   0.003   0.022
B T  4O27A                 74    0.173  24.023   0.558   0.970   0.001   0.030
B L  4O27A                 75    0.028   5.090   0.610   0.970   0.001   0.030
B V  4O27A                 76    0.054   8.346  -0.534   0.970   0.001   0.030
E A  4O27A                 77    0.320  35.231   0.876   0.923   0.002   0.076
B D  4O27A                 78    0.179  25.866   0.310   0.858   0.002   0.139
B L  4O27A                 79    0.047   8.551   0.806   0.502   0.002   0.495
E Q  4O27A                 80    0.450  80.406   0.478   0.321   0.003   0.675
E L  4O27A                 81    0.524  95.853   1.171   0.246   0.004   0.750
B I  4O27A                 82    0.093  17.113   0.699   0.053   0.005   0.942
E D  4O27A                 83    0.666  96.014   0.556   0.003   0.003   0.994
B F  4O27A                 84    0.189  37.892   0.113   0.109   0.005   0.886
E E  4O27A                 85    0.668 116.700   0.820   0.246   0.004   0.750
E G  4O27A                 86    0.256  20.147  -0.019   0.339   0.016   0.645
B K  4O27A                 87    0.276  56.855   0.238   0.622   0.015   0.363
E K  4O27A                 88    0.394  81.005   0.469   0.802   0.014   0.185
B D  4O27A                 89    0.246  35.420   0.434   0.923   0.002   0.076
B V  4O27A                 90    0.026   3.950   0.853   0.975   0.003   0.022
B A  4O27A                 91    0.094  10.381   0.449   0.992   0.000   0.007
E Q  4O27A                 92    0.280  49.990   1.568   0.992   0.000   0.007
B I  4O27A                 93    0.031   5.735   0.632   0.992   0.000   0.007
B F  4O27A                 94    0.048   9.553   0.790   0.988   0.000   0.012
B N  4O27A                 95    0.098  14.303  -0.073   0.970   0.001   0.030
B N  4O27A                 96    0.135  19.749   0.099   0.970   0.001   0.030
B I  4O27A                 97    0.036   6.586  -0.026   0.938   0.007   0.055
B L  4O27A                 98    0.070  12.817  -0.090   0.938   0.007   0.055
E R  4O27A                 99    0.348  79.715   0.737   0.717   0.014   0.269
E R  4O27A                100    0.290  66.295   0.874   0.561   0.047   0.393
E Q  4O27A                101    0.347  61.974   0.863   0.184   0.043   0.773
E I  4O27A                102    0.320  59.237  -0.416   0.115   0.016   0.868
E G  4O27A                103    0.715  56.310  -1.120   0.016   0.005   0.979
E T  4O27A                104    0.632  87.714  -1.623   0.016   0.005   0.979
E R  4O27A                105    0.562 128.675  -0.080   0.016   0.005   0.979
B T  4O27A                106    0.283  39.238   0.120   0.016   0.005   0.979
B P  4O27A                107    0.066   9.351  -0.069   0.113   0.043   0.844
B T  4O27A                108    0.100  13.870   0.383   0.453   0.248   0.299
B V  4O27A                109    0.074  11.451  -0.081   0.701   0.107   0.192
E E  4O27A                110    0.450  78.528   1.244   0.911   0.033   0.057
B Y  4O27A                111    0.144  30.794   1.208   0.879   0.010   0.111
B I  4O27A                112    0.032   5.920   0.380   0.879   0.010   0.111
B C  4O27A                113    0.187  26.199  -0.374   0.802   0.014   0.185
E T  4O27A                114    0.522  72.415   0.458   0.622   0.015   0.363
B Q  4O27A                115    0.205  36.613   1.409   0.181   0.016   0.803
B Q  4O27A                116    0.197  35.113   0.925   0.782   0.003   0.216
E N  4O27A                117    0.549  80.359   1.041   0.858   0.002   0.139
B I  4O27A                118    0.044   8.140   0.692   0.970   0.001   0.030
B L  4O27A                119    0.025   4.632   1.295   0.970   0.001   0.030
B F  4O27A                120    0.207  41.545   0.243   0.988   0.000   0.012
B M  4O27A                121    0.165  32.996   0.633   0.970   0.001   0.030
B L  4O27A                122    0.030   5.585   0.511   0.970   0.001   0.030
B L  4O27A                123    0.069  12.561  -0.700   0.923   0.002   0.076
E K  4O27A                124    0.410  84.378   0.977   0.782   0.003   0.216
B G  4O27A                125    0.182  14.300  -1.300   0.406   0.004   0.590
B Y  4O27A                126    0.118  25.259   0.433   0.176   0.004   0.820
E E  4O27A                127    0.609 106.392   0.060   0.053   0.005   0.942
E S  4O27A                128    0.302  35.359   0.341   0.058   0.017   0.925
E P  4O27A                129    0.389  55.171  -0.532   0.257   0.016   0.727
E E  4O27A                130    0.417  72.867  -0.764   0.339   0.016   0.645
B I  4O27A                131    0.068  12.635  -0.540   0.522   0.016   0.462
B A  4O27A                132    0.060   6.568  -1.571   0.660   0.049   0.291
B L  4O27A                133    0.150  27.538  -0.760   0.717   0.014   0.269
B N  4O27A                134    0.181  26.440  -0.354   0.717   0.014   0.269
B C  4O27A                135    0.041   5.770  -0.532   0.694   0.003   0.303
B G  4O27A                136    0.077   6.091  -1.274   0.694   0.003   0.303
B I  4O27A                137    0.141  26.067  -0.404   0.858   0.002   0.139
B M  4O27A                138    0.035   6.943   0.028   0.938   0.007   0.055
B L  4O27A                139    0.017   3.076   1.455   0.938   0.007   0.055
B R  4O27A                140    0.149  34.029   0.365   0.938   0.007   0.055
B E  4O27A                141    0.095  16.596  -0.848   0.879   0.010   0.111
B C  4O27A                142    0.025   3.538   0.504   0.802   0.014   0.185
B I  4O27A                143    0.038   7.104   0.121   0.622   0.015   0.363
E R  4O27A                144    0.391  89.585   0.971   0.339   0.016   0.645
B H  4O27A                145    0.184  33.433   0.648   0.430   0.016   0.555
E E  4O27A                146    0.434  75.820   0.666   0.622   0.015   0.363
E P  4O27A                147    0.411  58.321   0.567   0.717   0.014   0.269
B L  4O27A                148    0.046   8.514  -0.060   0.751   0.050   0.199
B A  4O27A                149    0.089   9.841   0.496   0.701   0.107   0.192
E K  4O27A                150    0.434  89.294   1.682   0.649   0.163   0.188
B I  4O27A                151    0.146  27.066   0.583   0.578   0.229   0.194
B I  4O27A                152    0.086  15.892   0.671   0.352   0.332   0.316
B L  4O27A                153    0.078  14.263  -0.433   0.148   0.418   0.435
E W  4O27A                154    0.393  94.396   0.327   0.064   0.216   0.721
E S  4O27A                155    0.407  47.677   0.026   0.184   0.043   0.773
E E  4O27A                156    0.755 131.881   0.099   0.600   0.003   0.397
E Q  4O27A                157    0.255  45.489   0.912   0.858   0.002   0.139
B F  4O27A                158    0.036   7.125   0.089   0.923   0.002   0.076
E Y  4O27A                159    0.296  63.255   0.844   0.938   0.007   0.055
E D  4O27A                160    0.414  59.715   1.577   0.938   0.007   0.055
B F  4O27A                161    0.052  10.356   0.949   0.938   0.007   0.055
B F  4O27A                162    0.050  10.055   0.612   0.879   0.010   0.111
E R  4O27A                163    0.462 105.844   1.611   0.879   0.010   0.111
B Y  4O27A                164    0.188  40.240   0.242   0.879   0.010   0.111
B V  4O27A                165    0.056   8.546   0.170   0.751   0.050   0.199
E E  4O27A                166    0.515  89.936  -0.524   0.455   0.046   0.498
B M  4O27A                167    0.254  50.745  -0.100   0.181   0.016   0.803
E S  4O27A                168    0.471  55.178  -0.810   0.115   0.016   0.868
B T  4O27A                169    0.168  23.274  -0.476   0.268   0.043   0.689
B F  4O27A                170    0.073  14.671   0.160   0.605   0.105   0.290
E D  4O27A                171    0.268  38.561   0.120   0.725   0.163   0.112
B I  4O27A                172    0.077  14.264   0.882   0.725   0.163   0.112
B A  4O27A                173    0.028   3.042   0.531   0.779   0.100   0.120
B S  4O27A                174    0.106  12.435  -0.144   0.879   0.010   0.111
E D  4O27A                175    0.274  39.426  -0.110   0.938   0.007   0.055
B A  4O27A                176    0.036   3.967  -0.900   0.923   0.002   0.076
B F  4O27A                177    0.088  17.702   0.159   0.970   0.001   0.030
E A  4O27A                178    0.411  45.270   0.868   0.970   0.001   0.030
B T  4O27A                179    0.128  17.781   0.400   0.970   0.001   0.030
B F  4O27A                180    0.037   7.406   0.142   0.988   0.000   0.012
E K  4O27A                181    0.329  67.737   1.389   0.988   0.000   0.012
E D  4O27A                182    0.282  40.679   0.096   0.970   0.001   0.030
B L  4O27A                183    0.049   9.027   0.338   0.970   0.001   0.030
B L  4O27A                184    0.061  11.151  -0.125   0.923   0.002   0.076
E T  4O27A                185    0.346  47.976  -0.275   0.782   0.003   0.216
E R  4O27A                186    0.371  84.867   0.559   0.502   0.002   0.495
B H  4O27A                187    0.147  26.685   0.257   0.109   0.005   0.886
E K  4O27A                188    0.408  83.987   0.635   0.782   0.003   0.216
E L  4O27A                189    0.643 117.678   0.395   0.879   0.010   0.111
B L  4O27A                190    0.101  18.493   0.420   0.879   0.010   0.111
B S  4O27A                191    0.033   3.868   0.003   0.938   0.007   0.055
E A  4O27A                192    0.286  31.506   0.514   0.970   0.001   0.030
E E  4O27A                193    0.537  93.866   1.284   0.970   0.001   0.030
B F  4O27A                194    0.107  21.415   0.501   0.970   0.001   0.030
B L  4O27A                195    0.052   9.576   0.279   0.970   0.001   0.030
E E  4O27A                196    0.436  76.204   0.945   0.923   0.002   0.076
E Q  4O27A                197    0.543  97.033   1.370   0.858   0.002   0.139
B H  4O27A                198    0.200  36.289   0.428   0.694   0.003   0.303
B Y  4O27A                199    0.076  16.284   1.367   0.923   0.002   0.076
E D  4O27A                200    0.563  81.085   1.385   0.970   0.001   0.030
E R  4O27A                201    0.374  85.623   1.970   0.970   0.001   0.030
B F  4O27A                202    0.027   5.479   0.735   0.988   0.000   0.012
B F  4O27A                203    0.062  12.504   1.062   0.988   0.000   0.012
E S  4O27A                204    0.528  61.917   1.320   0.988   0.000   0.012
E E  4O27A                205    0.273  47.746   1.900   0.988   0.000   0.012
B Y  4O27A                206    0.039   8.420   0.523   0.988   0.000   0.012
B E  4O27A                207    0.260  45.387   1.406   0.970   0.001   0.030
E K  4O27A                208    0.439  90.385   1.645   0.970   0.001   0.030
B L  4O27A                209    0.058  10.620   0.022   0.923   0.002   0.076
B L  4O27A                210    0.070  12.780   0.140   0.782   0.003   0.216
E H  4O27A                211    0.622 113.105   0.143   0.502   0.002   0.495
E S  4O27A                212    0.390  45.708  -0.075   0.109   0.005   0.886
E E  4O27A                213    0.666 116.403  -0.884   0.109   0.005   0.886
B N  4O27A                214    0.264  38.650  -1.014   0.181   0.016   0.803
B Y  4O27A                215    0.196  41.778   0.218   0.660   0.049   0.291
B V  4O27A                216    0.127  19.443   0.338   0.831   0.044   0.125
B T  4O27A                217    0.194  26.963   0.346   0.831   0.044   0.125
B K  4O27A                218    0.143  29.436   0.553   0.879   0.010   0.111
B R  4O27A                219    0.231  52.945   0.258   0.879   0.010   0.111
E Q  4O27A                220    0.308  54.991   0.069   0.802   0.014   0.185
B S  4O27A                221    0.068   7.993  -0.935   0.858   0.002   0.139
B L  4O27A                222    0.050   9.210   0.653   0.923   0.002   0.076
E K  4O27A                223    0.435  89.521   1.782   0.970   0.001   0.030
B L  4O27A                224    0.101  18.420   0.342   0.970   0.001   0.030
B L  4O27A                225    0.047   8.642   0.458   0.970   0.001   0.030
B G  4O27A                226    0.169  13.308  -0.968   0.970   0.001   0.030
E E  4O27A                227    0.399  69.775   1.097   0.970   0.001   0.030
B L  4O27A                228    0.092  16.918   0.684   0.938   0.007   0.055
B L  4O27A                229    0.094  17.248   0.015   0.782   0.003   0.216
B L  4O27A                230    0.235  43.029  -0.127   0.502   0.002   0.495
E D  4O27A                231    0.363  52.294   0.475   0.321   0.003   0.675
E R  4O27A                232    0.455 104.172   0.365   0.600   0.003   0.397
E H  4O27A                233    0.320  58.244  -0.606   0.782   0.003   0.216
B N  4O27A                234    0.132  19.354  -0.420   0.782   0.003   0.216
B F  4O27A                235    0.213  42.729   0.521   0.923   0.002   0.076
E T  4O27A                236    0.494  68.546   1.149   0.970   0.001   0.030
B I  4O27A                237    0.082  15.078   0.207   0.970   0.001   0.030
B M  4O27A                238    0.090  17.989   0.042   0.970   0.001   0.030
E T  4O27A                239    0.356  49.322   0.318   0.923   0.002   0.076
E K  4O27A                240    0.387  79.647   1.332   0.879   0.010   0.111
B Y  4O27A                241    0.111  23.806   0.388   0.802   0.014   0.185
B I  4O27A                242    0.115  21.349  -0.674   0.522   0.016   0.462
E S  4O27A                243    0.443  51.978  -0.832   0.181   0.016   0.803
E K  4O27A                244    0.373  76.788   0.641   0.053   0.005   0.942
E P  4O27A                245    0.487  69.134  -0.164   0.782   0.003   0.216
E E  4O27A                246    0.564  98.443   0.566   0.782   0.003   0.216
B N  4O27A                247    0.136  19.954   0.343   0.923   0.002   0.076
B L  4O27A                248    0.029   5.328   0.584   0.970   0.001   0.030
E K  4O27A                249    0.333  68.457   1.326   0.970   0.001   0.030
B L  4O27A                250    0.092  16.790   0.842   0.970   0.001   0.030
B M  4O27A                251    0.028   5.643   0.250   0.970   0.001   0.030
B M  4O27A                252    0.048   9.525   0.858   0.970   0.001   0.030
E N  4O27A                253    0.351  51.357   0.983   0.923   0.002   0.076
B L  4O27A                254    0.092  16.790   0.266   0.858   0.002   0.139
B L  4O27A                255    0.072  13.256  -0.393   0.694   0.003   0.303
E R  4O27A                256    0.442 101.104   0.396   0.321   0.003   0.675
E D  4O27A                257    0.391  56.285  -0.573   0.257   0.016   0.727
E K  4O27A                258    0.618 127.225   0.053   0.181   0.016   0.803
E S  4O27A                259    0.367  43.059  -0.851   0.181   0.016   0.803
E R  4O27A                260    0.413  94.508   0.663   0.191   0.086   0.723
E N  4O27A                261    0.373  54.593   0.297   0.148   0.418   0.435
B I  4O27A                262    0.065  12.025   0.699   0.169   0.612   0.219
B Q  4O27A                263    0.180  32.077   0.409   0.172   0.690   0.138
B F  4O27A                264    0.107  21.535   0.119   0.169   0.612   0.219
B E  4O27A                265    0.271  47.379   0.056   0.169   0.612   0.219
B A  4O27A                266    0.054   5.995  -0.751   0.159   0.516   0.325
B F  4O27A                267    0.096  19.247   0.256   0.079   0.592   0.329
B H  4O27A                268    0.229  41.637   0.023   0.087   0.683   0.230
B V  4O27A                269    0.053   8.223  -0.273   0.018   0.846   0.136
B F  4O27A                270    0.075  14.992   0.108   0.018   0.846   0.136
B K  4O27A                271    0.166  34.043   0.803   0.011   0.918   0.071
B V  4O27A                272    0.029   4.396   0.857   0.011   0.918   0.071
B F  4O27A                273    0.048   9.714   0.848   0.011   0.918   0.071
B V  4O27A                274    0.042   6.502  -0.087   0.018   0.846   0.136
B A  4O27A                275    0.102  11.273  -0.572   0.022   0.552   0.426
B N  4O27A                276    0.202  29.631   1.163   0.004   0.138   0.858
E P  4O27A                277    0.585  82.997  -0.129   0.005   0.045   0.951
E N  4O27A                278    0.669  97.927  -0.157   0.005   0.015   0.979
E K  4O27A                279    0.416  85.468   0.192   0.016   0.005   0.979
B T  4O27A                280    0.224  31.083  -0.212   0.109   0.005   0.886
E Q  4O27A                281    0.652 116.394   1.032   0.858   0.002   0.139
E P  4O27A                282    0.417  59.130   1.507   0.858   0.002   0.139
B I  4O27A                283    0.022   3.996   0.908   0.923   0.002   0.076
B L  4O27A                284    0.200  36.693   1.159   0.970   0.001   0.030
E D  4O27A                285    0.562  81.027   2.008   0.970   0.001   0.030
B I  4O27A                286    0.067  12.377   0.926   0.970   0.001   0.030
B L  4O27A                287    0.041   7.580   0.294   0.975   0.003   0.022
E L  4O27A                288    0.423  77.433   1.302   0.938   0.007   0.055
E K  4O27A                289    0.552 113.546   1.557   0.858   0.002   0.139
B N  4O27A                290    0.167  24.390   0.021   0.406   0.004   0.590
B Q  4O27A                291    0.195  34.773   0.873   0.923   0.002   0.076
E A  4O27A                292    0.653  71.983   0.981   0.970   0.001   0.030
E K  4O27A                293    0.299  61.587   2.092   0.970   0.001   0.030
B L  4O27A                294    0.019   3.497   1.354   0.970   0.001   0.030
B I  4O27A                295    0.145  26.751   1.134   0.988   0.000   0.012
E E  4O27A                296    0.554  96.749   1.937   0.970   0.001   0.030
B F  4O27A                297    0.082  16.417   0.920   0.970   0.001   0.030
B L  4O27A                298    0.052   9.430   0.530   0.923   0.002   0.076
E S  4O27A                299    0.603  70.636   1.175   0.858   0.002   0.139
E K  4O27A                300    0.678 139.382   1.454   0.694   0.003   0.303
B F  4O27A                301    0.054  10.858   1.026   0.321   0.003   0.675
E Q  4O27A                302    0.551  98.426   0.465   0.109   0.005   0.886
E N  4O27A                303    0.406  59.482  -0.352   0.053   0.005   0.942
E D  4O27A                304    0.725 104.530  -1.135   0.016   0.005   0.979
E R  4O27A                305    0.429  98.264  -0.190   0.053   0.005   0.942
E T  4O27A                306    0.482  66.867  -1.056   0.058   0.017   0.925
E E  4O27A                307    0.592 103.440  -0.560   0.115   0.016   0.868
E D  4O27A                308    0.333  47.928  -0.617   0.181   0.016   0.803
E E  4O27A                309    0.678 118.499   0.355   0.339   0.016   0.645
E Q  4O27A                310    0.473  84.424   1.188   0.430   0.016   0.555
B F  4O27A                311    0.076  15.213   0.288   0.622   0.015   0.363
E N  4O27A                312    0.495  72.468   0.863   0.879   0.010   0.111
E D  4O27A                313    0.591  85.206   0.707   0.923   0.002   0.076
B E  4O27A                314    0.256  44.758   0.264   0.970   0.001   0.030
B K  4O27A                315    0.173  35.566   0.763   0.970   0.001   0.030
E T  4O27A                316    0.451  62.512   0.901   0.970   0.001   0.030
E Y  4O27A                317    0.337  72.081   1.784   0.970   0.001   0.030
B L  4O27A                318    0.047   8.661   0.795   0.988   0.000   0.012
B V  4O27A                319    0.088  13.556   0.493   0.988   0.000   0.012
E K  4O27A                320    0.521 107.129   1.719   0.970   0.001   0.030
E Q  4O27A                321    0.307  54.741   1.206   0.970   0.001   0.030
B I  4O27A                322    0.094  17.409  -0.743   0.923   0.002   0.076
E R  4O27A                323    0.558 127.874   0.930   0.782   0.003   0.216
E D  4O27A                324    0.758 109.228   1.301   0.502   0.002   0.495
E L  4O27A                325    0.541  99.057  -1.223   0.016   0.005   0.979

Explain the output.  Go back.
```

---
